# Mathematical difficulties as decoupling of expectation and developmental trajectories

**DOI:** 10.3389/fnhum.2014.00044

**Published:** 2014-02-06

**Authors:** Janet F. McLean, Elena Rusconi

**Affiliations:** ^1^Division of Psychology, School of Social and Health Sciences, Abertay UniversityDundee, UK; ^2^Department of Neurosciences, University of ParmaParma, Italy; ^3^Department of Security and Crime Sciences, University College LondonLondon, UK

**Keywords:** mathematical difficulties, mathematical development, children, school curriculum, intervention

## Abstract

Recent years have seen an increase in research articles and reviews exploring mathematical difficulties (MD). Many of these articles have set out to explain the etiology of the problems, the possibility of different subtypes, and potential brain regions that underlie many of the observable behaviors. These articles are very valuable in a research field, which many have noted, falls behind that of reading and language disabilities. Here will provide a perspective on the current understanding of MD from a different angle, by outlining the school curriculum of England and the US and connecting these to the skills needed at different stages of mathematical understanding. We will extend this to explore the cognitive skills which most likely underpin these different stages and whose impairment may thus lead to mathematics difficulties at all stages of mathematics development. To conclude we will briefly explore interventions that are currently available, indicating whether these can be used to aid the different children at different stages of their mathematical development and what their current limitations may be. The principal aim of this review is to establish an explicit connection between the academic discourse, with its research base and concepts, and the developmental trajectory of abstract mathematical skills that is expected (and somewhat dictated) in formal education. This will possibly help to highlight and make sense of the gap between the complexity of the MD range in real life and the state of its academic science.

There has been increasing interest in *mathematical difficulties* (MD) particularly as government departments seek to understand why countries such as the US and the UK have low levels of functional numeracy. For example, Gross et al. (2009) found that around 25% of those able to work in the UK do not have essential mathematical skills, and Parsons and Bynner (2005) reported that those with poor numeracy were twice as likely to be unemployed as those with competent levels. These low levels of attainment have been linked with the developmental disability dyscalculia where low mathematics achievement stands against a background of otherwise normal skills (e.g., language, memory, visuo-spatial attention, etc.), and is characterized as a primary impairment of number skills (Butterworth, 2005, 2010). However it seems unlikely that dyscalculia alone can account for the findings as its prevalence rates range between 1.3 and 10.3% (Devine et al., 2013). It seems thus likely that a large proportion of those with poor numeracy would instead have MD, which we theorize encompass a range of mathematical learning shortcomings that may manifest at various developmental stages and originate in a variety of underlying causes. To explore at what point in time these diverse difficulties may impact on mathematic ability, this review will explore what children are expected to learn as they go through the mathematics curriculum. Clarity on the “expectation trajectory” should prove extremely useful in a research field where no comprehensive and consensus model of a “developmental trajectory” is yet available. We will then identify a recent model that has outlined the basic cognitive components involved in mathematical skill development. This model lends itself naturally to identifying individual causes of MD, especially when difficulties are conceptualized as a decoupling between developmental and expectation trajectory at different stages in formal education.

## Defining MD

Although most people, when asked, will report having struggled with mathematics at some point in their lives, objective difficulties with the learning of mathematics are said to present when mathematical achievement is significantly lower than the average obtained by the appropriate age group. Official figures of attainment seem to suggest that in the UK 10% of children in formal education do not reach the required standards by ages 7 and 11 (DfES, 2012), and in the US 18% of children in formal education do not reach the required standards by ages 10 and 14 (National Center for Education Statistics, 2011; see also *Mathematics Curriculum* section). Individual achievement can be measured against consensus ideal standards (e.g., Common Core Standards for Mathematics, accessed August 9 2013, http://www.corestandards.org/Math/Practice), overall class achievement, or standardized tests. The latter are often based on academic and/or pedagogical models of mathematical cognition (e.g., Wide Range Achievement Test-Revised (WRAT-R; Jastak and Wilkinson, 1984; Woodcock Johnson-Revised (WJ-R) Calculation and Applied Problems subtests; Woodcock and Johnson, 1989).

**Figure 1:**
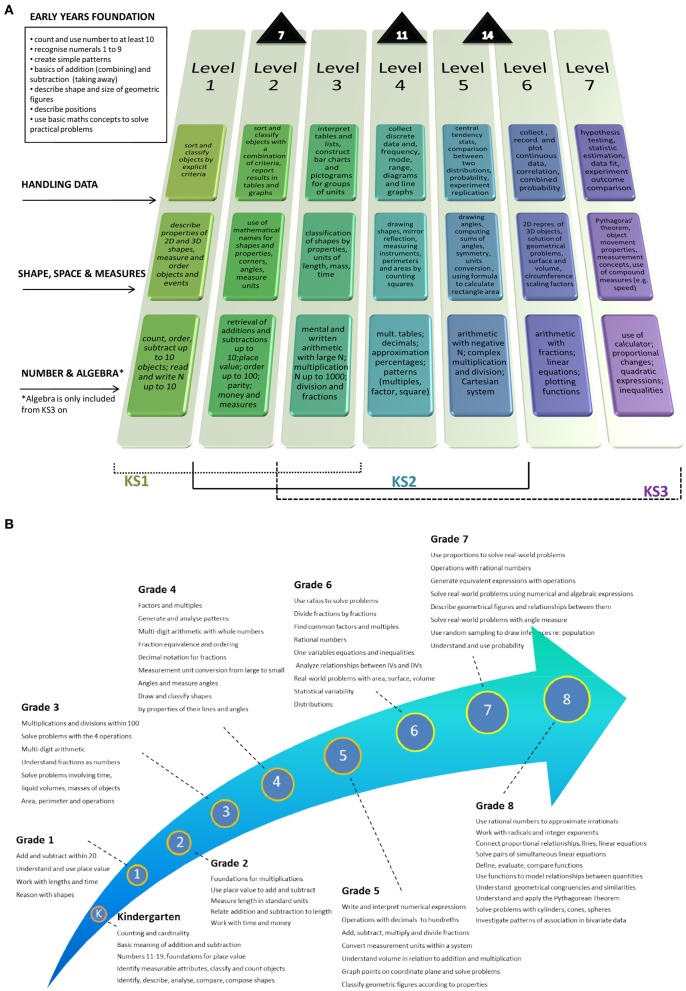
**(A)** Expectation trajectory with attainment targets for Number and Algebra, Shape, Space, and Measures, and Handling Data from the UK curriculum. On average, children reach Level 2 at age 7, Level 4 at age 11 and Level 5–6 at age 14. **(B)** Expectation trajectory with targets by grade from the Core Standards.

In developmental research children are typically selected as MD from a single assessment of their mathematical ability. One problem with selecting children this way is that there is little consensus about the method of selection. Some have included children who show a discrepancy between IQ and mathematics performance (e.g., Lindsay et al., 2001), but more commonly researchers have applied a cut-off criterion where children who perform below a given percentile on a standardized measure of mathematics achievement are defined as having MD (e.g., Geary et al., 2000; Butterworth, 2003; Szucs et al., 2013). Despite the popularity of a cut-off selection there is little consensus about at what level the cut-off criterion should be set and this can lead to differing cognitive profiles emerging from different research studies (Murphy et al., 2007). Another problem with selection based on a single assessment is that mathematics requires a range of different skills and these skills are different depending upon the child's expected stage of their mathematical development, so the same MD label may in principle indicate very different profiles. For example, MD at Grade 1 could indicate inability to use place value and perform simple additions and subtractions, whereas a classification of MD at Grade 5 could either indicate inability to translate numerical information into a Cartesian framework and solve geometrical problems or still indicate lack of more basic skills such as fluency in arithmetic operations (see e.g., Figure 1). In other words, inclusion in a MD group can be a reflection of these different stages of their understanding. The tests used for screening may differ for different age groups to reflect the expected stage of their development and so within-cohort differences are mostly meaningful in relation to age of the children and curriculum standards. Moreover, approximately 30% of individuals who are classified as having some sort of MD at any one time will not remain in the same category (i.e., they will not be classified as MD at further testing) over time (Silver et al., 1999; Mazzocco and Myers, 2003). Repeated testing however is extremely infrequent in practice and only a few targeted longitudinal studies have been conducted so far (e.g., Geary et al., 2000; Jordan et al., 2002, 2003; Vukovic and Siegel, 2010).

A host of persistent and or temporary factors have been proposed as the underlying cause of MD but no universal consensus risk assessment, prognostic and rehabilitative model is available as yet. Severe MD are known to be associated with psychological, neurological, and genetic conditions, such as epilepsy, Turner's syndrome, fragile X syndrome, phenylketonuria and ADHD (Shalev et al., 2000). Furthermore MD are often co-morbid with delayed language development and behavioral disorders (e.g., Manor et al., 2001). In cases where MD are co-morbid with other learning difficulties or pathological conditions, it is very difficult to discriminate whether the mathematics impairment is a primary deficit or whether it is instead due to a deficit in other functions. But even in cases where MD do not seem to be associated with any other conditions and a clear achievement-potential discrepancy is found in otherwise non-problematic individuals, several potential mechanisms may be at play. Despite this, no empirically-based normative developmental trajectory for mathematics learning has been yet established, and the non-problematic range of variation for age-appropriate levels is currently untested (see also Szucs and Goswami, 2013).

## Mathematics curriculum

It is thus informative to take a closer look at template “expected developmental trajectories” of recent formulation, setting the standard against which individual or cohort performance will be contrasted within and between schools in a near future. Amount of tolerance toward performance deviance from the standards will probably depend on school-specific pedagogical and curriculum choices and on the average achievement levels of the cohort involved. Professional diagnoses of MD, often based on standardized tests will then typically follow in the most severe cases and dedicated support staff may be called in. It is however important to bear in mind that difficulties with math are unlikely to receive the same support as language difficulties, due to the only relatively recent awakening of public awareness (e.g., Bynner and Parsons, 1997). In this section we provide an overview of the guiding principles and targets behind the mathematics curriculum for England and the US and explore the impact that curricula can have on MD by setting age-appropriate targets.

We have chosen to provide descriptions of the English and US curriculum as both governments have developed very clear standards. For those interested in a comparative perspective, The Trends in International Mathematics and Science Study (TIMSS) has made some comparisons on curricula in many different countries but it is beyond the scope of this review to outline each of these and we thus redirect the reader to the latest report (TIMSS 2011 International Results in Mathematics, accessed 20 September 2013, http://timssandpirls.bc.edu/timss2011/downloads/T11_IR_Mathematics_FullBook.pdf). We will first describe two different curricula and compare their approach to learning this multi-layered skill. We look at what each child is expected to have achieved by the end of each level or grade, thus showing what cognitive components may impact at different stages of mathematics development. This discussion comes at a critical time as the national curriculum for mathematics in England has just completed its consultation period at the Department for Education and is due to be implemented in September 2014. The US is already in the stages of implementing a new curriculum and it is currently under adoption in 45 states.

### England

In England, all children in state funded schools are measured on their academic progress at 4 stages in their school career (approximately age 7, 11, 14, and 16). These are known as Key Stages 1–4 (KS1-4). Mathematics is a compulsory national curriculum subject at all 4 key stages (see Table 1; a full description of the curriculum can be found at http://www.education.gov.uk/schools/teachingandlearning/curriculum/primary/b00199044/mathematics). Assessment within the first three Key Stages is measured in levels. A child can reach one of 3 different levels of achievement (i.e., typically Level 1–3 for Key Stage 1, Level 4–6 for Key Stage 2 and Level 5–7 for Key Stage 3, although there may be overlaps such as children leaving KS 1 at Level 4 or only achieving Level 3 at the end of KS 2), ranked in ascending order of skill complexity (see Figure 1A). In addition, mathematics is included for 3–5 year old children within the Early Years Foundation Stage (EYFS). This review concentrates on this EYFS, as well as KS 1 and 2 as this encompasses the ages (3–11 years) where most research has focused on MD, however a brief description of KS3 is included as this includes math skills that some exceptional children in primary school can work toward.

**Table 1:** **Breakdown of Key Stages in the English curriculum per school year and chronological age**.

**Chronological ages**	**School years**	**Key stage**
3–5	Pre-school/reception	EYFS
5–7	1–2	1
7–11	3–6	2
11–14	7–9	3
14–16	10–11	4

At the EYFS, children are introduced to mathematics through guidelines set out in the Problem Solving, Reasoning, and Numeracy framework (DCSF, 2008). Within this framework, there are skills outlined for using numbers as labels and for counting (e.g., using number names accurately, counting up to four and beyond and recognizing numerals); calculating (e.g., using the vocabulary involved in adding and subtracting, understanding “more” and “less” to compare two numbers, relating addition to combining two groups and subtraction to “taking away”); and shapes, spaces, and measures (e.g., using language to compare quantities, talk about, recognize and recreate simple patterns, using words to describe position). Both formative and summative assessment of these skills is recorded in each child's Early Learning Profile. Early years practitioners are encouraged to use play as part of the child's learning activities and the focus is on providing the basic skills necessary to make the transitions into KS1.

At all stages there is a general attainment target for using and applying mathematics. However this does not have detailed standards and is included to ensure that teachers instruct students about the connections between different areas of mathematical knowledge (National Curriculum for England Mathematics, 1999, p. 6). The skills measured at KS1 come under two broad sections called number and shape, and space and measures. Under each section the curriculum outlines a number of standards that set out detailed targets. For example, within the numbers section, the target for counting states that “Pupils should be taught to count reliably up to 20 objects at first and recognize that if the objects are rearranged the number stays the same; be familiar with the numbers 11–20; gradually extend counting to 100 and beyond” (National Curriculum for England Mathematics, 1999, p. 16). The target for number patterns and sequences is to “create and describe number patterns” and use this knowledge to make predictions. This includes patterns of multiples of 2, 5, and 10, sequences of odd and even numbers and the relationship between halving and doubling (National Curriculum for England Mathematics, 1999, p. 16). At KS1, academic performance is assessed via individual teacher assessment against the National Curriculum Attainment Targets rather than by examination and pupils is expected to achieve KS1 level 2. The latest government figures were published in 2011 and they show that 90% of children were achieving the expected level.

At KS2 the skills measured are number; shape, space, and measures, handling data, and mental arithmetic. Again each section has standards with associated targets. For example, the target for counting states that “Pupils should be able to count on and back in tens or hundreds from any two- or three-digit number; recognize and continue number sequences formed by counting on or back in steps of constant size from any integer, extending to negative integers when counting back” (National Curriculum for England Mathematics, 1999, p. 21). Within the handling data, the targets for processing, representing and interpreting data include “interpreting tables, lists and charts used in everyday life; constructing and interpret frequency tables, representing and interpreting discrete data using graphs and diagrams (National Curriculum for England Mathematics, 1999, p. 27). Pupils at KS2 have formal assessments in the final year of primary school and this provides information about the children's math performance before they move onto secondary schooling and KS 3 and 4. Pupils are expected to reach a KS2 Level 4 standard in mathematics and schools were set a target to ensure 60 per cent of pupils achieve this standard (DfES, 2010). In 2011, the percentage of pupils attaining level 4 or above at KS2 was 84% (DfES, 2012). Whilst this may seem a high level, there were still a substantial number of schools with attainment below the 60% target that suggests that many children are not achieving the necessary skills in mathematics before they progress to secondary school education.

The skills measured at KS3 come under the same broad topic headings as at KS2. As expected, the level of difficulty and range of skills required increases. For example, within the handing data topic children are expected to move up to a level of understanding where they can use statistical calculations and begin to use probability. Again each section has standards with associated targets. Similar to KS1, pupils are assessed through teacher assessment and pupils are expected to reach either KS3 Level 5 or 6 in mathematics (National Curriculum for England Mathematics, 1999, p. 7). In 2011, the percentage of pupils attaining level 5 or above at KS2 was 81% (DfES, 2012). A more detailed representation of the attainment targets at each level with Key stages 1–3 is presented in Figure 1A; using and applying mathematics is not included in this figures as there the attainment targets are not as detailed as the other sections.

### United states

In contrast to the English system, the mathematics curriculum in the US has been largely up to individual states (before introduction of the new curriculum); there was no common curriculum. Nevertheless performance on these diverse curricula has been assessed by the National Assessment of Educational Progress (NAEP) at Grades 4 (age 9–10 years). In 2011, a nationally representative sample of 209,000 children from 21 urban districts of the US were assessed on five content areas: number properties and operations (e.g., computation with or understanding of whole numbers and common fractions and decimals), measurement (e.g., knowledge of units of measurement for capacity, length, area, volume and time), geometry (e.g., knowledge and understanding of simple shapes, and relationships between shapes such as symmetry and transformations), data analysis, statistics, and probability (e.g., understanding data collection and organization, reading and interpreting representations of data, and basic concepts of probability), and algebra (e.g., understanding of algebraic representation, patterns, and rules; graphing points on a line or a grid; and using symbols to represent unknown quantities). This found that 82% of pupils were classed as at or above basic in mathematics, which suggests that by age 9 years, 18% of children experience some form of difficulty learning mathematics. Furthermore, a similar assessment conducted with over 175,000 eighth-graders (age 13–14 years) found that the percentage at or above basic levels drops to 73% (National Center for Education Statistics, 2011). Thus it appears that the number of children with difficulties increases as they progress through the curriculum. Although it is not possible to compare performance between the English and US children because the measures of assessment differ considerably, as well as the ages of the children, it is clear that a significant number of children in both countries are not achieving attainment targets in mathematics. Furthermore, statistics collected at later stages of schooling show that performance drops further as children progress through their schooling and gets decoupled from the expectation trajectory in about 20–30% of the children (National Center for Education Statistics, 2011).

Given that the evidence suggests that there is decoupling, it is worth describing here that a new Common Core State Standards Initiative (National Governors Association Center for Best Practices, Council of Chief State School Officers, 2010) has recently proposed a math curriculum that is to be adopted by the majority of states from 2014 (see Figure 1B). This curriculum lays out the mathematics content that should be learned at each grade level from kindergarten to Grade 8 (see http://www.corestandards.org/Math for a full description of the curriculum). Educators in the US and elsewhere have found it necessary to redefine what students should be able to understand and do when learning mathematics. They defined common core standards, while recognizing that the assumption that what is learnt before should determine what is learnt at a later stage is unwarranted, given the current state of the science. At the moment, indeed, only partial models of learning pathways to mathematical concepts and skills can be obtained from scientific and education research, with very few exceptions (see e.g., LeFevre et al., 2010). The criteria for the standards were developed from academic research; analyses of which skills are required of students entering college and workforce training programs and by looking at standards from high achieving nations and data from the TIMSS in collaboration with some of the teaching bodies within the US. For the purpose of this review, which concentrates on children up to age 11, we will report the four key domains: Operations and Algebraic Thinking; Number and Operations in Base 10; Measurement and Data; Geometry. In addition Counting and Cardinality is included for Kindergarten and Number and Operations is included for Grades 3 and 5. Within each domain, there are several standards, clustered into related standards. For example, during Kindergarten, within the domain of Counting and Cardinality, children are expected to acquire number names and the count sequence sufficiently to count up and determine the number of objects in a set and to compare numbers; within Operations and Algebraic thinking they should understand the concept of addition as putting together and adding to, and subtraction as taking apart and taking from; within Number and Operations in Base 10 they should be able to work with numbers up to 19 and begin to understand place value; within Measurement and Data, they should be able to describe and compare measurable attributes such as length or weight, and classify objects and count the number of objects in categories; and within Geometry, they should be able to identify and describe shapes such as squares, triangles and circles as well as analyze, compare, create, and compose shapes (National Governors Association Center for Best Practices, Council of Chief State School Officers, 2010; Common Core Standards for Mathematics, p. 10). The first assessments of this new curriculum are due to begin in the 2014–2015 school year.

One of the guiding teaching principles made explicit by the Core Standards is teachers' focus on “mathematical understanding” as the royal pathway, along with procedural learning, to meaningful achievement. “There is a world difference between a student who can summon a mnemonic device to expand a product such as (a + b) (x + y) and a student who can explain where the mnemonic comes from. [The latter] may have a better chance to succeed to a less familiar task such as expanding (a + b + c) (x + y).” (Common Core State Standard Initiative for Mathematics, 2010, p. 4)

Note that the proposed assessment is tightly connected with this definition of “understanding.” If assessment is only focused on the ability to reach and provide the correct solution to a given problem, it will often confound procedural or mechanic learning with mathematical understanding. Specifically assessing mathematical understanding means assessing: (1) the ability to generalize knowledge to novel situations, (2) the ability to explain the underlying meaning of procedures.

- Difficulty with math is not only defined by an inability to follow the procedure but may unveil a deeper problem (i.e., a lack of mathematical understanding)
- To assess learning, we needn't focus exclusively on achievement: apparently normal achievement at one stage, may still lead to later difficulties with numbers, if it is exclusively driven by procedural learning
- Difficulties may appear at a later stage due to lack of proper understanding at earlier stages; a child who shows MD at Grade 7 may not have understood concepts from Grade 5 and 6 despite normal achievement.

To illustrate how development is thought to develop, Figure 1B shows the trajectory of US children following the new Common Core State Standards curriculum.

## Comparing curricula

Both the current English and new US curricula provide clear and detailed targets for arithmetic development and how children will build up an understanding of this complex discipline, and there are many commonalities between them. For example, they both have a strong focus on counting and place value within the early years and use this skill as a basis for progression onto calculations. Calculations are conducted first with single digit and then multi-digit numbers. They both also have a focus on shapes, space, and measurement that begins in early years and is included at all levels of the curriculum. However there are also differences that may impact on the selection of MD children. The US curriculum is much more strongly focused on number and operations within the early grades and purposely does not introduce additional topics until later schooling (The Hunt Institute, 2011). The idea behind this was to achieve an intricate grounding in these skills which can then be taken forward to new skills later in the trajectory. There is also more emphasis on conceptual understanding than rote procedural learning. The English curriculum does introduce these other topics. For example it has a strong emphasis on patterns. Even at KS1, children are expected to be able to “create and describe number patterns” whereas within the US curriculum patterns are not included as a standard until Grade 4 (approximately 9–10 years of age). Another contrast is that the English curriculum outlines the use of mental models for calculation. The target is to develop rapid recall of number facts and procedures; a target which calls directly on memory processes.

## Expectation trajectory and implied cognitive skills

In the remainder of this article we will provide an overview of one of the mathematical development models that, in our opinion, looks promising for the identification of potential causes and areas of intervention in MD. These can occur at any stage of primary schooling when there is a decoupling between the general expectation and an individual's actual developmental trajectory.

Although our knowledge on mathematical learning and cognition has enormously expanded in the last few decades, there is no consensus or comprehensive developmental trajectory for mathematical skills, let alone a consensus model on MD. Grade placements for specific topics are therefore suggested on the basis of national and international comparisons, educators' collective experience, and researchers' and mathematicians' professional judgment. By establishing a standard set of principles and objectives on such basis, the initiative opens to the possibility of improving the process on a large scale as research on learning and effectiveness progresses.

The expected developmental trajectory, based on a consensus between education professionals (Figures 1A,B) rather than academic research output and theoretical models, will thus continue to set the main standard against which a given individual or cohort will contrast their performance and will be deemed as having MD or not in the years to come. The amount of tolerance toward deviance from the standards (and thus criteria for MD) is likely to be influenced by school-specific pedagogical and curriculum choices and also on the average achievement levels of the cohort involved. Once the most severe cases are identified as such, professional diagnoses of MD will then be typically undertaken and dedicated support staff may be called in—although difficulties with mathematics are unlikely to receive the same support as language difficulties (Butterworth et al., 2011).

The most straightforward type of MD (or MD risk) diagnosis is probably the one done at the earliest stages such as Kindergarten and Grade 1, where although multiple cognitive skills are already interacting to enable numerical understanding, such understanding is still very far removed from of the level of abstraction expected in later years. Typically diagnosis even at this early stage follows from a child's poor performance on a standardized test of mathematics especially in comparison to performance on measures of other abilities such as reading or IQ (e.g., Geary et al., 2000, 2007; Murphy et al., 2007; Chu et al., 2013), although other studies suggest that screening using experimental measures of number sense such as approximate number system acuity or skills in several counting tasks may be suitable (e.g., Jordan et al., 2006, 2009; Chu et al., 2013). At this stage abstraction is very much rooted in and inferred from concrete experiences such as those outlined above in the Kindergarten Common Core standards. These imply very basic and foundational skills, part of which may rest on a core number processing toolkit and basic cognitive abilities we share with animals (see e.g., Gallistel, 1989; Butterworth, 1999; Kawai and Matsuzawa, 2000; Dehaene, 2001). Part already rests on an interaction with symbolic processing skills and independent functions such as language and spatial processing (see e.g., Jordan et al., 2009; Cirino, 2011). It is apparently on these very concrete and relatively simple foundations that abstract mathematics starts being taught and learnt. In following years and up to the adult stage, with increasing abstraction the picture becomes much more complex and difficult to decipher.

**Figure 2:**
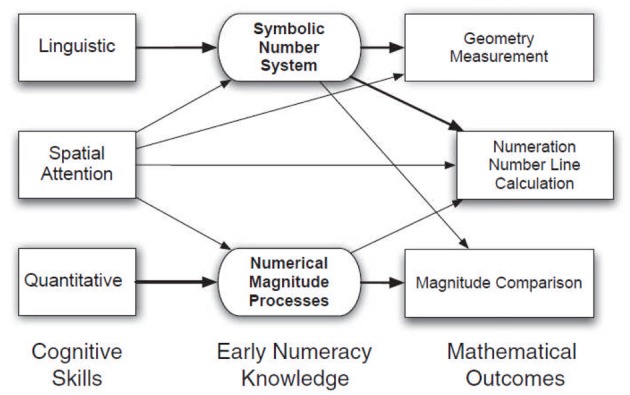
**Schematic of the Pathways model with predicted relations among cognitive precursors, early numeracy skills, and mathematical outcome measures.** From LeFevre et al. (2010) Child Development. With permission. Note this is Canadian.

LeFevre et al. (2010) have recently proposed a model including multiple cognitive factors that may contribute to the developmental trajectory and determine an individual's mathematical outcomes throughout developmental stages. Their model is based on the triple-code neuropsychological model of adult numerical processing (Dehaene et al., 2003), one that has collected the widest consensus and empirical support in recent years. LeFevre et al.'s (2010) model provides a simple and promising framework that could be especially well suited to identify cognitive precursors that may become important to fulfill expectations at different stages of the developmental trajectory up to adult age. No doubt, such framework would benefit from further refinements and the inclusion of possible additional cognitive precursors (see Figure 2). However the model does lend itself well to the translation of academic concepts into educational targets by providing a theoretically-driven framework to evaluate and predict achievement targets. Moreover, by suggesting developmental pathways that are compatible with the guiding assumptions of much research in adult numeracy, knowledge will be easier to update and predictions about potential neural substrates could also be more easily derived from adult studies.

## A useful working framework

LeFevre et al. (2010) have provided an initial test of their model in a longitudinal study with a large cohort of children from preschool and Kindergarten (aged between 4; 5 and 6; 6 years), following their progress in mathematics for 3 years. They hypothesized that linguistic, quantitative, and spatial attention pathways contribute independently to number skills and that they vary in their unique and relative contributions to mathematical outcomes, depending on task demands. Their test for basic quantitative knowledge (a cognitive precursor of more complex numeracy knowledge) was an object counting task with small sets of objects, and they used subitizing speed as a summary index (i.e., the speed in correctly recognizing numerosities from 1 to 3). Subitizing is generally considered a reasonable index of children's quantitative knowledge (see e.g., Landerl et al., 2004) although a visuo-spatial short-term memory component may also be at play (Feigenson et al., 2004). Linguistic skills were assessed via measures of vocabulary and phonological awareness (Dunn and Dunn, 1997; Wagner et al., 1999); and spatial attention skills were measured with an adaptation for children of the spatial span task (aka Corsi blocks test; see Passolunghi and Cornoldi, 2008). Each of these indexes, therefore, captured a complex of skills rather than a single element in relation with language, quantity (or numerosity) and space, while still maintaining some level of specificity.

As a measure of early numeracy skills, LeFevre et al., used the number of correct responses in single and multi-digit number naming from Arabic format and the percentage of correct trials in a non-linguistic arithmetic task on small quantities (e.g., mental operations between sets of objects; see Levine et al., 1992). These tasks are meant to maximally tap on either the linguistic or the quantity code (see Figure 2). Finally, as measures of mathematical outcomes at Grade 2, both standardized and more experimental tests were used: the Numeration, Geometry and Measurement subtests from the KeyMath Test-R (Connolly, 2000) and the Calculation tests from the WJ Tests of Achievement-R (Woodcock and Johnson, 1989) covering most of the skills required by Grade 1 and 2, a Number Line task (Laski and Siegler, 2007) requiring to place numbers in the appropriate position on a line whose extremes are labeled as 0 and 1000 and taken as a measure of coordination between symbolic and quantitative knowledge, and a comparison task between single digit numbers whose physical size was orthogonally varied with their numerical size, tapping on symbolic but especially quantitative knowledge (Landerl et al., 2004; Holloway and Ansari, 2009).

The thickness of the connectors in Figure 2 between cognitive precursors (left-hand boxes) and numerical knowledge (central boxes) indicates the relative importance of the contribution of cognitive precursors to early numeracy skills measures as they emerged from a multiple regression analysis. Measures of linguistic skills predicted up to 30% of the variance in the symbolic number system task, whereas subitizing latency predicted up to 32% of the variance in the non-linguistic arithmetic task. Neither of them predicted performance in the alternative number task. Spatial attention was apparently involved in both the symbolic and the magnitude task (predicting 16 and 15% of the variance respectively). Three different factors, corresponding to the linguistic, spatial, and quantitative pathways, were entered in a further multiple regression analysis to assess their predictive power on the standardized and experimental mathematical outcomes (right-hand boxes). Overall they accounted for a substantial proportion of variability (26–56%) in both the conventional and the experimental outcome measures. As shown by connector thickness, the relative contribution of each pathway varies with the outcome considered.

The linguistic pathway (i.e., individual measures of vocabulary and phonological awareness and number naming) contributed to all mathematical outcomes, but especially those related with geometry and measurement, numeration and calculation (i.e., classical tests of school achievement) and the Number Line task. The spatial attention pathway resulted involved in all outcomes, except for the experimental magnitude comparison task, whereas the quantitative pathway was found to contribute to magnitude comparison, numeration, number line and calculation but not to geometry and measurement.

**Figure 3:**
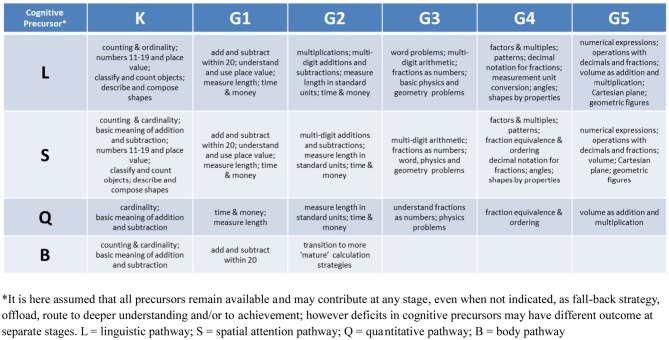
**Hypothetical coupling of expectation and developmental trajectory.** Targets for Kindergarten and the first five grades have been associated with their most likely cognitive precursors on the basis of an expanded Pathways model (see LeFevre et al., 2010 and our section “Expectation Trajectory and Cognitive Skills”).

In summary, most mathematical outcome measures in the LeFevre et al. (2010) study, including standardized batteries, depend on the functioning of the symbolic number system, with a heavily linguistic component. Whether the symbolic number system may itself be related to the quantitative pathway was assessed with a more complex quantitative task, non-symbolic arithmetic by Gilmore et al. (2010). They suggested that “children's non-symbolic numerical abilities […] appear to contribute to their achievement in mathematics primarily because they are associated with children's successful learning of number words and symbols, which figures prominently in […] the kindergarten mathematics curriculum and the assessment of mathematical learning […]” (Gilmore et al., 2010; p. 8). Furthermore, Gilmore et al. (2010), had partialled out the effect of literacy achievement and verbal intelligence. On the whole, these findings would suggest that primary impairments in the use of the symbolic system (Ansari, 2008) and/or linguistic deficits (Manor et al., 2001) will exert a pervasive negative effect on individual trajectories of mathematical achievement. For example, in the UK curriculum the rudiments of an abstract symbolic number system beyond 10 (i.e., beyond the number of fingers, typically used as concrete and intuitive representations for both cardinality and ordinality) constitute attainment targets at Level 2. Thus the developmental trajectory of pupils with impairment in the symbolic pathway could start diverging at about age 7 and become more and more decoupled from the expectation trajectory throughout schooling. The quantitative pathway alone, indeed, will become progressively inadequate to handle the abstraction of concepts and complexity of skills expected in later years (e.g., numbers of increasing size, mental arithmetic with two and three digit numbers, decimals, recognition of pattern in the number series, negative number arithmetic, linear and quadratic equations). Under the curriculum proposed by the Standards, difficulties would emerge even earlier, between Kindergarten and Grade 1 (i.e., ages 5 and 6), due to the early introduction of place value, with two-digit numbers and operations between them (see also Figure 3). MD from impairment in the symbolic system at age 5 in the US might therefore be diagnosed as MD at age 7 in the UK. Based on adult models of the role of language in number processing, specific impairments in the language system will particularly compromise the learning of rote memory arithmetic, and in particular multiplications and complex mental operations (Dehaene et al., 2003). Major difficulties will thus start to emerge when pupils reach US Grade 3 or UK attainment Level 3 (i.e., around the age of 8), particularly if children do not spontaneously discover alternative strategies to verbal representations. Teachers may also teach alternative strategies.

What could be considered as core numerosity processing (see e.g., Butterworth, 2010) or analogue magnitude processing (Dehaene et al., 2003), and whose selective impairment is thought to underlie developmental dyscalculia (e.g., Rubenstein and Henik, 2009) does not appear to contribute as importantly to tasks in the mathematics curriculum tapping geometry and measurement. In the presence of a problem in the quantitative system, achievement tests involving these tasks may therefore be relatively spared when compared to tasks of numeration, calculation, number comparison etc. from the earliest age. In a recent training study, Park and Brannon (2013) reported a relation between adult performance improvements in tasks tapping the approximate number system (a likely quantitative precursor), and performance improvements in corresponding symbolic arithmetic tasks. This connection between an approximate number system and mathematics proficiency would seem also to be domain-specific, and Dewind and Brannon (2012) showed that improvement in a comparison task involving the approximate number system did not generalize to a homologue visuo-spatial task. This calls the prediction of developmental dissociations between difficulties arising from impairments at the level of non-verbal, non-symbolic quantity processing and impairments in auxiliary domains—even if conceptually related with quantity, such as spatial processing—with the latter exerting more subtle and elusive effects on the developmental trajectory of number processing as opposed to measurement and geometry skills.

In LeFevre et al.'s (2010) study, acomplex measure of spatial attention and working memory was found to contribute to geometry and measurement, and all other outcome measures, except for symbolic magnitude comparison with single digits. Purely attentional deficits may therefore compromise most achievement tasks except for those primarily resting on the core quantitative pathway. MD will thus be more subtle than with those deriving from impairment in the symbolic and quantitative pathways, yet equally spread across attainment targets from UK Level 2 or US Grade 1, when place value and visuo-spatial geometric properties are taught. Additional strain may be put on the system when working toward UK Level 5 (or at US Grade 5), with the introduction of the Cartesian system and connections between number, geometry, and measurement. It is however also possible that this may in fact provide a novel and affordable method to parse space, thus improving these children's performance in mathematics. An interesting possibility that would need to be explored with *ad hoc* empirical studies.

It may be surprising that there is not an independent pathway for working memory within this framework as many researchers have found that performance on working memory measures can specifically predict mathematics performance (e.g., Holmes and Adams, 2006; Bull et al., 2008). Indeed research that has specifically explored the impact of working memory subsystems (phonological loop, visuo-spatial sketch pad (VSSP), and central executive) in the longitudinal development of mathematical learning has suggested that the VSSP may be important for younger children (e.g., Bull et al., 2008; Holmes et al., 2008; Simmons et al., 2008). One suggestion is that young children's mental representations of quantities rely heavily on visual-spatial representations, as they have not yet developed a spontaneous verbal rehearsal system. As children progress through school they increasingly use verbal representations of quantities such as number words and the role of the VSSP has less impact (see Rasmussen and Bisanz, 2005). One reason for apparent missing pathway is that LeFevre et al.'s description differs from others. For example, many studies have included variations of the Corsi Blocks task as their measure of visuo-spatial working memory. LeFevre et al., also used a version of this task but describe it as a spatial attention task due to problems distinguishing the nature of the task. Nevertheless LeFevre et al., note that a more detailed account about the role of working memory in mathematical learning may be necessary. In particular they suggest that working memory may play an important role in integrating knowledge from the linguistic and quantitative pathways.

More recently LeFevre et al.'s (2010) basic architecture was used as a working framework by Cirino (2011) who maintained the original conceptual distinctions but expanded the range of tasks (symbolic vs. non-symbolic) used to measure the effects of quantity precursors in Kindergarten on a single outcome measure (i.e., small written sums). Interestingly, the symbolic (with a strong linguistic element) vs. non-symbolic distinction between precursors of later mathematical outcomes was also highlighted by Jordan et al. (2006, 2009), who reported how children's socio-economic status defined by their family income level interacts with the symbolic/linguistic pathway (but also see Mejias and Schiltz, 2013). That is to say children from low-income families will enter primary school with an initial disadvantage due to poorer start-up symbolic/linguistic resources despite showing in most numerical tasks (e.g., verbal and non-verbal counting, verbal and non-verbal arithmetic, estimation, number patterns) similar growth trajectories as children from high-income families, with the notable exception of verbal story problems. Therefore, given the pervasive effects that the symbolic number processing pathway may exert on later mathematical outcome (see also Jordan et al., 2002), and their characterization of MD as difficulty in story problems and arithmetic combinations], children from low socio-economic backgrounds should be considered at higher risk for MD and intervention strategies could be specifically devised from Kindergarten. This point has also been corroborated more recently by Gilmore et al. (2010) who highlighted two factors contributing to mathematics achievement: a non-symbolic aptitude, which is essentially insensitive to differences in socio-economic status, and symbolic ability that may be responsible for the higher achievement levels found in association with higher socio-economic status. They point out that preschool exposure to conventional symbol systems is higher in higher for children of wealthy families (Jordan et al., 1992; Griffin and Case, 1996), therefore the achievement gap due to impoverished symbolic environment may be eliminated by targeted interventions at Kindergarten (Siegler and Ramani, 2008). Interestingly, such interventions may also provide useful evidence for the putative causal link between symbolic skills and the developmental trajectory of mathematics learning, which as of now can only be two co-varying variables due to the correlational character of the evidence reported above.

It has also been shown that reading difficulties predict lower number skills especially those implying verbal sequential factors, and should therefore be treated as risk factors too (Jordan et al., 2006). Manor et al. (2001) established a relation between developmental language disorders and measures of mathematical outcomes. In particular, both receptive and expressive language impairments were associated with low scores in reasoning principles and arithmetic operations. Only expressive deficits, however predicted poor performance in counting principles. Despite the sometimes different theoretical frameworks adopted by different research groups, especially regarding whether a single number core processing module or domain general skills are at the origin of difficulties with mathematics (see e.g., Bull et al., 2008; Locuniak and Jordan, 2008; Desoete et al., 2009; Geary et al., 2009), data suggest that mathematics difficulties at Kindergarten will persist and predict an atypical growth rate in the following years (Morgan et al., 2009). This is not to say that MD cannot appear after an otherwise normal developmental trajectory in successive years or a child who has experienced difficulties since Kindergarten cannot benefit from interventions at a later stage (the predictive power of early mathematics difficulties on later difficulties never reaches 100%, and MD can also appear at later stages).

## Adding new components to the original framework

As previously mentioned, the merit of LeFevre et al.'s (2010) model consists of bringing together in a simple but comprehensive framework the main cognitive modules that are expected to interact and inform mathematical outcomes across the normal developmental trajectory, rather than focusing on one single cognitive domain. It does so by paralleling a well-known neuropsychological model of adult mathematical cognition (Dehaene et al., 2005). Specifics about the component pathways and their operationalization's (e.g., whether approximate quantities and numerosity processing should be considered as partially independent subcomponents of the quantity pathway) can be improved by testing predictions and expanding the model's evidence base. Interesting connections with MD can be established by classifying groups of individuals (e.g., children with Williams syndrome vs. children with spina bifida vs. dyslexics) based on the pathway that may be most problematic. With the model, MDs characterized by different patterns of development-expectation decoupling and educational outcomes may be diagnosed and assessed (e.g., LeFevre et al., 2010). To this purpose, and based on the literature on both adult and developmental number processing, we would like to suggest that additional components may be useful to create a model to diagnose/assess/predict MD.

For example in addition to spatial and linguistic precursors, body representations may be related to numeracy skills, as suggested by the interactions between finger gnosis and number processing in both adults and children. In a few studies, MD was reportedly associated with impairments of finger gnosis, left-right orientation and writing, also known under the name of developmental Gerstmann syndrome (see e.g., Miller and Hynd, 2004). In the past, this was typically taken as evidence for a functional connection between all of these abilities and between finger gnosis and the development of number skills in particular (see e.g., Butterworth, 1999). The connection seems to be corroborated by the fact that acquired brain lesions localized to the left posterior hemisphere often produce the adult version of Gerstmann's syndrome (a cluster of neuropsychological symptoms characterized by left–right confusion, agraphia, acalculia, and finger agnosia; Gerstmann, 1940). Likewise, TMS studies have identified contiguous neural substrates in adult participants with causal effects on numerical processing, finger gnosis, and categorical left–right discrimination (Rusconi et al., 2005; Hirnstein et al., 2011). Without undermining the significance of these associations, Kleinschmidt and Rusconi (2011) have recently suggested that the Gerstmann functions, including finger gnosis and calculation may indeed be supported by a network of cortical regions in the left posterior parietal lobe whose intraparietal projections converge toward a common subcortical bottleneck location. A small and localized lesion to the bottleneck location will cause systematic association of symptoms. The adult version of Gerstmann's syndrome would thus be characterized as an anatomical syndrome, meaning that the four symptoms may not be functionally interdependent, and yet still subjected to the very same local neural efficiency parameters and maturation constraints. This may also suggest an anatomical mechanism for the somewhat elusive developmental version of Gerstmann syndrome and provide an additional cluster of non-numerical predictors—although not necessarily cognitive precursors - of mathematics achievement, that could help identify a neurofunctional locus for certain patterns of MD.

At least a transient phase of finger counting and finger calculation almost invariably precedes the mature mathematical cognition in the developmental trajectory, although educators may have different views on its utility (e.g., Moeller et al., 2011). In fact, the use of fingers to represent number is ubiquitous across ages and cultures (Dantzig, 1954; Butterworth, 1999). Children use finger counting as an initial strategy to understand and keep track of counting and calculate, even if this is often seen as just a very primitive strategy (Geary et al., 2007). Amputees and children with congenital agenesia of hands and fingers use phantom fingers as quantifiers (Poeck, 1964). Finger counting strategies also tend to be used by older children and adults with MD, to make up for deficient mental number representations. Furthermore, performance in tests of finger gnosis before formal schooling selectively predict mathematical outcomes at a later age (e.g., Fayol et al., 1998; Noël, 2005) and it has been reported that early finger training may improve numerical abilities at a later stage (Gracia-Bafalluy and Noël, 2008; but see Fischer, 2010). According to a very popular idea, these latter findings are consistent with numerical knowledge being represented together with the same sensory and motor features that are engaged during learning (see e.g., Fischer, 2012). There is indeed empirical evidence that traces of finger counting habits influences—not necessarily always in a beneficial way—symbolic number representations and calculation processes (Domahs et al., 2008, 2010; also see Fischer and Brugger, 2011) for a review on other relevant interactions). Another possibility is that the crosstalk between numerical and body representations is not integral to numerical representations but provides a means to offload and free working memory resources while processing numerical information in a task-dependent way (e.g., Fischer, 2006). Of relevance to this context, however, are not the exact mechanisms underlying the cross-talk between fingers and numbers and whether traces of finger processing are indeed integral to the numerical representation. The consensus and empirical evidence that finger counting does play a role in the development of numerical skills could thus suggest a useful expansion and improve the predictive power of the LeFevre et al. (2010) model by including a dedicated body representation component amongst the cognitive precursors.

In addition, as noted above, although working memory is included in the LeFevre et al. (2010) framework as part of spatial attention pathway, an additional or more detailed component may be required to address the more complex aspects of mathematical development. For example, LeFevre at al. note that working memory may be involved in the coordination of information from the world and from memory. The central executive is usually considered the working memory subsystem responsible for coordinating information, including controlling attentional resources (Baddeley, 2003). There have been many studies which have shown that children with MD show impaired performance compared to typically developing children on tasks which are designed to tap into central executive processes (e.g., Bull et al., 1999; Geary et al., 2004; McLean and Hitch, 1999). However the role of the central executive within mathematical development is less well understood. Recently, LeFevre et al. (2013) examined executive attention, which they suggest encompasses executive functioning and the central executive in working memory, in children's development of mathematics. Children completed executive attentional tasks and mathematical tasks (specifically tasks on knowledge of the number system and arithmetic fluency) at either 8 or 9 years of age. They repeated the mathematical tasks 1 year later. Using structural equational modeling, LeFevre et al., showed that executive attention was concurrently predictive of both knowledge and fluency but predicted growth in performance only for fluency. LeFevre et al., conclude that the executive functioning may be particularly important in the early years of mathematical development when new tasks are being taught and learned. We would also expect that executive functioning, rather than being a cognitive precursor, may play a crucial role in integrating knowledge from the linguistic and quantitative pathways.

In Figure 3 we show how the model could be used to draw predictions on the cognitive abilities that are necessary at each developmental stage as specified in the Core Standards. This in turn will suggest what cognitive problems may subtend MD at different stages.

## Interventions

One may assume that precise knowledge of the mechanism(s) underlying an individual's difficulties with mathematics may be a prerequisite for devising tailored teaching, remediation and intervention strategies. Therefore it is important to have a well-defined model that can encapsulate where difficulties may occur and how remediation can pinpoint these difficulties.

All researchers and educators agree that mathematic competence is not a single well-defined skill but encompasses a range of skills. What is clear from the expectation trajectory is that low attainment, particularly measured at single assessment, can also reflect a single or multitude of difficulties with mathematical concepts. The evidence suggests that although there may be around 2–10% of the population with the severe specific difficulty dyscalculia, it is also likely that the 15–20% described in the Parsons and Bynner (2005) report have difficulties with only certain aspects of mathematics. These difficulties may be sufficient to hinder their education and employment prospects. Of course there may be other reasons for low achieving population such as math anxiety (e.g., Ashcraft, 2002) or poor teaching. However it is often difficult to disentangle these from poor attainment. In addition, as noted above, some problems may be due to co-morbid developmental disorders such as dyslexia or ADHD (Rubenstein and Henik, 2009). Nevertheless even those with co-morbid conditions will have difficulties which impact at different stages of mathematic development.

Thus the question remains on the best way to assist those who do have underlying difficulties. In a series of well designed empirical studies, Fuchs and colleagues (see Fuchs et al., 2009, 2013; Powell et al., 2009; Powell and Fuchs, 2010) developed and tested the effects of extensive training of children with MD on targeted foundational skills, for example counting or retrieval, on typical math achievement tasks (e.g., Number Combinations). They derive the rationale for their interventions (which are also available commercially as the software Pirate Math) from existing empirical evidence linking specific foundational skills with more complex math and curriculum targets. Dowker (2009) noted that there has been an increase in the number of intervention programs as the government and charities highlight problems in numeracy. Dowker is very clear in her recommendations that any intervention should be individualized to reflect the fact that math is a multi-layered skill and difficulties can occur at different stages. Recent reviews of the efficacy of interventions for students who are showing signs of struggling with numeracy (e.g., Kroeger et al., 2012), have tried to assess a selection of the current range of interventions available and suggest future directions. Kroeger et al. (2012) evaluated 20 commercially available programs (mostly available in the US) by exploring whether each program was developed from neuroscientific research, what cognitive processes were targeted by the program, and the kind of research that supported the program. They explicitly implemented this approach because they believe that the most effective intervention practices would integrate research from neuroscientists and cognitive developmental psychologists as well as math educators. In particular it has been shown that the impact of neuroscientific data can influence the general public perception of research, including interventions, as brain research appears more compelling than behavioral data (Weisberg et al., 2008). However, for an intervention to be deemed successful it must build on evidence from all three fields.

Kroeger et al. (2012) found that only three programs included publisher-reported use of neuroscience research in their development, and here they focused on the triple code model (Dehaene et al., 2003). These were Fluency and Automaticity through Systematic Teaching with Technology (FASTT Math), Number Worlds (NW), and The Number Race (NR). In addition only FASTT Math, NW and NR plus two others (Accelerated Math (AM), Corrective Mathematics (CM) were supported by empirical, peer-reviewed research on their efficacy. Their review concluded that although 4 of these 5 intervention programs showed improvements on test scores, the programs emphasize representation of number sense, akin to the quantitative pathway, math facts and working memory. For example, in the NR, quantitative pathways and math facts are trained. Children play a computer game that requires them to first carry out a numerical comparison task; they must choose the larger of two quantities of treasure faster than a competitor. The competitor is essentially the computer program represented by a character on the screen, and the difference in magnitude between the two quantities can be large or small to manipulate difficulty. Furthermore the quantity can be represented in a non-symbolic format, sets of gold pieces, in symbolic Arabic numerals or symbolic number words. Presenting the numerical information in different ways is designed to strengthen links between representations of number (Wilson et al., 2006). At a higher level of difficulty, the quantities can only be worked out on completion of arithmetic problems (e.g., is 6–2 bigger than 4 + 0?). On completion of the comparison task, the game moves counting task. The set of treasure they chose in the comparison task is placed next to treasure from a competitor. The child then races against their competitor by moving the same number of squares in grid as they have pieces of treasure. This is done by counting each piece of treasure one at a time and hence loading on one-to-one correspondence and cardinality. Kroeger et al.,note that few intervention programs have focused on problem solving or executive function although the CM program may load onto both of these as it attempts to teach students rules and strategies to help solve arithmetic problems. Executive function is potentially one cognitive process that underlies mathematical understanding and, in general, it appears that apart from number sense there is little intervention targeting the underlying cognitive processes. To mesh with our expectation trajectory—intervention could be targeted where a difficulty is found.

Another commercially available intervention, which has been developed in the UK and not included in Kroeger et al., is Catch Up Numeracy. In this intervention program children are individually assessed and provided with targeted sessions building on their strengths and weaknesses. This is low intensity intervention program and children complete just two 15 min remediation sessions a week. However it has shown some good improvements with children who have weaknesses in their math performance but are not necessarily dyscalculic. For example, Holmes and Dowker (2013) showed that children identified as MD who followed the Catch Up program for 30 weeks showed significant gains in their numeracy. These gains were twice as large as other children with MD who had received no intervention and more than the gains expected from typically developing children.

One of the interventions mentioned here, the Number Race (http://www.thenumberrace.com), is freely available and is very explicitly connected with the adult neuropsychological model that also shaped LeFevre et al.'s (2010) framework. More recently the researchers have added another game to develop fluency in arithmetic, the Number Catcher (http://www.thenumbercatcher.com/). Peer reviewed research is not available for the Number Catcher but research in the Number Race suggests effects on core numerical processing. For example, Räsänen et al. (2009) tested 30 preschoolers who had been identified as having poor numeracy skills and compared them to 30 typically developing children. Half the children followed a software program called Graphogame-Math that trains children to compare small numerical differences; and half the children played the Number Race games. After 3 weeks of playing the games 10–15 min a day, both experimental groups demonstrated improved performance in number comparison but did not improve in other number skills such as verbal and object counting. The developers thus suggest that their programs should be used in conjunction with other techniques. Both the Number Race and Number Catcher do make use of game software to engage children with mathematics. This application of games for an educational purpose, or gamification, is becoming an increasingly more popular way to motivate learners (e.g., Deterding, 2012).

In summary, there is growing demand and concurrent development of interventions for math difficulties. There is some evidence to suggest that intervention can improve scores on mathematics tests but also a warning that the intervention is not targeted sufficiently at the underlying cognitive skills of mathematics nor designed for individuals who may show differing profiles of difficulties. Future interventions should draw upon the growing body of evidence that mathematic difficulties can occur at different stages and for different underlying reasons. Some existing intervention programs might eventually lead to significant improvements in mathematical understanding if the program attempts to pinpoint specific skills that are required for mathematical competence. That should be attuned to different cognitive pathways and combination of skills at every developmental stage (e.g., Figure 3) and should be conducted within a theoretically-driven framework. In this way, applied can also be used to feed back into theory and contribute with new knowledge toward the delineation of an empirically-based developmental trajectory.

## Author contributions

The authors contributed equally to the writing of this paper. The order of authorship is arbitrary.

### Conflict of interest statement

The authors declare that the research was conducted in the absence of any commercial or financial relationships that could be construed as a potential conflict of interest.
